# The Role of Periostin in the Occurrence and Progression of Eosinophilic Chronic Sinusitis with Nasal Polyps

**DOI:** 10.1038/s41598-017-08375-2

**Published:** 2017-08-25

**Authors:** Ming Xu, Daishi Chen, Haojie Zhou, Weiwei Zhang, Jun Xu, Lei Chen

**Affiliations:** 1 Department of Otorhinolaryngology head and neck surgery, Chinese PLA General Hospital, Beijing, 100083 China; 2 Ningbo Diagnostic Pathology Center, Ningbo, 315021 China; 3 Department of Otorhinolaryngology, The Affiliated Hospital of The Medical School of Ningbo University, Ningbo, 315020 China

## Abstract

Chronic rhinosinusitis with nasal polyps (CRSwNP) is a highly heterogeneous disease with different host defence responses. However, whether periostin and vascular endothelial growth factor (VEGF) are similarly impaired in patients with eosinophilic CRSwNP (ENP) and those with non-eosinophilic CRSwNP (nENP) remains unclear. We sought to evaluate the expression and possible modulation of periostin and VEGF, regulated on activation normal T expressed and secreted (RANTES) and eotaxin-2 in the polyp tissues from 30 patients with ENP and from 36 patients with nENP and in middle turbinate tissues from 12 control subjects. We found that ENP tissues exhibited a significantly increased expression of periostin and VEGF compared with tissues from patients with nENP and control subjects (*P* < 0.05, respectively). Accordingly, the expression of VEGF, RANTES, and eotaxin-2 in ENP fibroblasts was significantly up-regulated after stimulation with up-regulated periostin *in vitro*, but the expression of VEGF and RANTES was significantly inhibited by stimulation with down-regulated periostin. Our findings suggest that periostin might play an important role in the occurrence and progression of ENP and might be a potential therapeutic target.

## Introduction

Chronic rhinosinusitis with nasal polyps (NP) is a heterogeneous group of sinus disorders^1^. NP is thought to be classified into a number of subtypes according to clinicohistological features. NP with eosinophil infiltration in the nasal polyps have been classified as eosinophilic chronic rhinosinusitis with nasal polyps (ENP) versus non-eosinophilic chronic rhinosinusitis with nasal polyps (nENP)^2^, each is characterized by distinct degrees of severity, prognoses, and therapeutic strategies^3^. The clinical characteristics of ENP are apparently different from those of nENP, and a high eosinophilic infiltration in the polyps predicts a worse outcome and a higher risk for polyp recurrence after surgical treatment^4^. It is important to understand the underlying disease processes of different subtypes of NP because the different inflammatory subtypes are clinically relevant based on their potential to have different responses to therapeutic interventions^5^.

Periostin is a recently characterized matricellular protein belonging to the fasciclin family that interacts with several integrin molecules (α_v_β1, α_v_β3 and α_v_β5) on cell surfaces, providing signals for tissue development and remodeling^6^. It has been found that periostin is a highly inducible product of IL-4 or IL-13, signature cytokines of Th2-type immune responses^7, 8^. Evidence has suggested that periostin accelerates eosinophil recruitment and activation and is involved in allergic inflammation and sustaining eosinophil-mediated inflammation^9^. RANTES and eotaxin-2, members of the CC family of chemokines, are particularly significant to eosinophils recruitment and infiltration during NP formation^10, 11^. However, the mechanism by which eosinophils are selectively recruited in nasal polyps remains to be clarified, and whether periostin might influence the tissue eosinophilia of ENP by modulating eotaxin-2 and RANTES secretion is not clear and warrants further investigation. Meanwhile, vascular endothelial growth factor (VEGF), a 45-kDa, heparin-binding homodimeric glycoprotein, is a major inducer of angiogenesis and capillary permeability^12^. VEGF plays a significant role in the regulation of remodeling in NP, which is characterized by tissue remodeling and an edematous nasal mucosa^13^. Evidence suggests that periostin could promote angiogenesis by upregulating VEGF through the focal adhesion kinase (FAK) or the signal-regulated kinase 1/2 (ERK 1/2) mediated signaling pathways^14, 15^. To date, some investigators have proposed that periostin is produced locally in the sinonasal tissues and might contribute to polyp formation^16, 17^; meanwhile, we had found that patients with ENP were more likely to be atopic^18^, so we speculate that periostin might play an important role in tissue remodeling in NP via inducing VEGF, RANTES and eotaxin-2 production. However, current evidence for the relationships among VEGF, RANTES, eotaxin-2 and periostin and various nasal disorder subtypes is scarce, and their importance in different subtypes of NP pathogenesis is not well understood.

Therefore, in the present study, we investigated and compared the expression of VEGF and periostin in nasal tissue from patients with ENP and nENP subtypes to evaluate whether host defenses are similarly impaired. Meanwhile, we assessed the stimulatory effect of periostin on VEGF, RANTES and eotaxin-2 expression in NP-derived fibroblasts (NPDFs) and further investigated whether the occurrence and progression of ENP are associated with periostin-induced VEGF, RANTES and eotaxin-2 up-regulation.

## Methods

### Patients and tissue samples

The methods were carried out in accordance with the CONSORT 2010 guideline, including any relevant details. All subjects or their parents provided written informed consent before enrolment in the study. This study was approved by the Ethics Committee of the Affiliated Hospital of the Medical School of Ningbo University, Ningbo, China.

Between April 1, 2014, and July 31, 2015, patients with NP and controls were recruited from the Affiliated Hospital of the Medical School of Ningbo University. NP was diagnosed according to the criteria of a European position paper on rhinosinusitis and nasal polyps from the European Academy of Allergology^19^. The inclusion criteria for NP patients included bilateral nasal polyps and the presentation of ≥2 symptoms including congestion, anterior/posterior drip, facial pain/pressure, reduction or loss of smell for at least 3 months, mucopurulent discharge from the middle meatus, oedema/mucosal obstruction primarily in the middle meatus, and mucosal changes within the ostiomeatal complex and/or sinuses. Patients who suffered antrochoanal polyps, cystic fibrosis, fungal sinusitis (mycetoma, allergic fungal sinusitis), and primary ciliary dyskinesia were excluded from this study.

A total of 66 NP patients were included in this study, and 12 cases with head and facial trauma (HFT) without a history of chronic rhinitis were included as control subjects. Oral glucocorticoid and intranasal steroid sprays were discontinued at least 3 months and 1 month before the test, respectively. The demographic data of all subjects enrolled in this study are listed in Table 1. Nasal tissue was obtained from all subjects during surgery. NP tissues (approximately 0.5 g) were collected from the apex region of polyps in NP patients, and the non-polyp tissues (approximately 0.5 g) were isolated from the middle turbinates of controls. All NP cases were classified as ENP or nENP cases according to the percent of eosinophils in tissues, with 10% used as the cut-off value^2^.

**Table 1:** Subjects’ characteristics.

	Control subjects	ENP	nENP
No. of patients	12	30 (45.45%)	36 (54.55%)
Sex, male/female	9/3	24/6	19/17
Age (y)	32.5 (21–43)	44.5 (25–71)	34.6 (16–63)
Duration (y)	—	3.4 (1–8)	5.1 (2–21)
Asthma history, yes/no	—	4/26	0/36
Smoking, yes/no	—	21/9**	9/27
Eosinophilis (%)		19.92 ± 9.19 (10.8–41.2)	4.97 ± 2.22 (1.5–9.0)

Age and duration are presented as medians (ranges). **P <0 .01 v.s. nENP.

### Reagents

The polyclonal rabbit anti-periostin (ab14041) and anti-VEGF (ab46154) antibodies were purchased from Abcam International Inc. (Cambridge, UK). The anti-eotaxin-2 and anti-RANTES antibodies were purchased from Biosource International, Inc. (Camarillo, CA, USA). TNF-α was purchased from Shanghai Jin’an Biological Technology, LTD (Shanghai, China).

### Histologic staining

Paraffin sections (4 µm) were used for histology. In this study, hematoxylin and eosin (HE) staining was used to evaluate submucosal capillary vessels in 10 randomly selected high-power fields (hpfs; x400 magnification) under light microscopy in a blinded manner.

Immunohistochemical (IHC) staining was performed using the peroxidase-labelled streptavidin-biotin technique. Briefly, 4-µm-thick sections were deparaffinized by serial treatment. After blocking endogenous peroxidase with 3% hydrogen peroxide and 1% BSA, the sections were incubated with anti-periostin (1:100) or anti-VEGF (1:100) at 4 °C overnight. Thereafter, each section was incubated with a secondary antibody for 30 min at room temperature, followed by a further 30 min with a horseradish peroxidase–labelled streptavidin complex (Zhongshanjinqiao, Beijing, China). The distribution of peroxidase was visualized by incubating the sections in a solution containing 3% 3,3-diaminobenzidine tetrahydrochloride before counterstaining with hematoxylin and covering the section with a cover slip. Negative control staining was obtained by replacing the primary antibodies with IgG at the appropriate concentrations. Periostin and VEGF staining intensity on histologic sections was scored according to the intensity of the dye colour and the number of positive cells in a blinded manner. The intensity of the dye colour was graded as 0 (no colour), 1 (light yellow), 2 (light brown), or 3 (brown), and the number of positive cells was graded as 0 (<5%), 1 (5–25%), 2 (25–50%), 3 (50–75%) or 4 (>75%). The immunohistochemical reaction was scored by multiplying the percentage of positive cells by their prevalent degree of staining and were assigned to one of 4 levels: a score of 0 (−), scores of 1-scores (+), scores of 5–8 (++), and scores of 9–12 (+++)^20^.

### Real-time quantitative reverse transcription polymerase chain reaction (PCR)

The mRNA expression levels of periostin and VEGF were evaluated using quantitative polymerase chain reaction (qPCR) analysis. Briefly, the tissues were washed twice with cold PBS, and RNA was extracted using TRIzol reagent (Invitrogen) following the manufacturer’s protocol. cDNA was generated with Oligo-dT primers. The primers were designed using Primer 5 and the Oligo 6.0 designer program as follows: periostin: forward, 5′-GGAATTCGCCACCATGATTCCCTTTTTACCCATG-3′; reverse, 5′-GCTCTAGATCACTGAGAACGACCTTCCCT-3′; VEGF: forward, 5′-GCCTCGCCTTGCTGCTCTACC-3′; reverse, 5′-CACACTCCAGGCCCTCGTCATTG-3′; and GAPDH (housekeeping gene): forward, 5′-GGAGATTGTTGCCATCAACG-3′; reverse, 5′-TTGGTGGTGCAGGATGCATT-3′. The reaction components were 0.2 μl cDNA, 2.5 μl 10 × Taq enzyme buffer, 1 μl 10 mol/L dNTP, 0.2 μl forward and reverse primers, and 0.2 μl Taq enzymes. Water was added to a final volume of 25 μl. The PCR conditions were as follows: a denaturing step of 95 °C for 2 min; then a cycle of 94 °C for 1 min, 56 °C for 1 min, and 72 °C for 1 min and 15 s; and a final extension step of 75 °C for 5 min. The PCR was performed with Illumina Eco Real-time PCR using a 2^−ΔΔCt^ method.

### Western blot analysis

Briefly, the cells were collected, and the total cellular protein was extracted in 100 μl of RIPA lysis buffer at 4 °C for 30 minutes. The protein concentrations were determined using the Bradford method. Samples containing 10 mg of protein were boiled, subjected to SDS-PAGE in 10% tris-glycine gels, and electrophoretically transferred to polyvinylidene fluoride membranes. The membranes were incubated with 5% fat-free skim milk in a tris-buffered saline solution containing 0.05% Tween-20 for 1 hour at room temperature and then incubated with the primary antibodies of rabbit polyclonal anti-periostin (1:200, Abcam International Inc. Cambridge, UK), VEGF (1:200) overnight at 4 °C. The membranes were then incubated with a horseradish peroxidase-linked secondary antibody and finally processed using an ECL chemiluminescence reaction kit (Cell Signaling, Danvers, MA), followed by exposure on medical film. The band density of the target protein relative to GAPDH was quantified using the Bio-Rad Quantity One 1-D Analysis Software (Bio-Rad Laboratories, Hercules, CA).

### Isolation and induction of NPDFs and control tissue-derived fibroblasts

Primary fibroblasts were randomly collected from 10 patients with NP (ENP = 5, nENP = 5) and from 5 cases with middle turbinate tissues from control subjects by using enzymatic digestion to establish the *in vitro* cell cultures. Briefly, the obtained tissues were washed three times with antibiotics containing Hank’s Balanced Salt Solution (HBSS) and were cut into small pieces (1 mm^3^). The tissue explants were treated with 0.5% trypsin for 7 minutes at 37 °C, followed by neutralization with serum-containing DMEM-F12. After washing three times with HBSS, the explants were plated into 6-well culture dishes (Cell BIND Surface, Corning, NY) and cultured with DMEM medium and 10% FBS in a 37 °C, 5% CO_2_ humidified incubator for approximately 2 weeks. When the primary culture reached confluence at 70–80%, the nasal fibroblasts were sub-cultured into 10-cm dishes.

### Cell transfection assay

**Figure 1:**
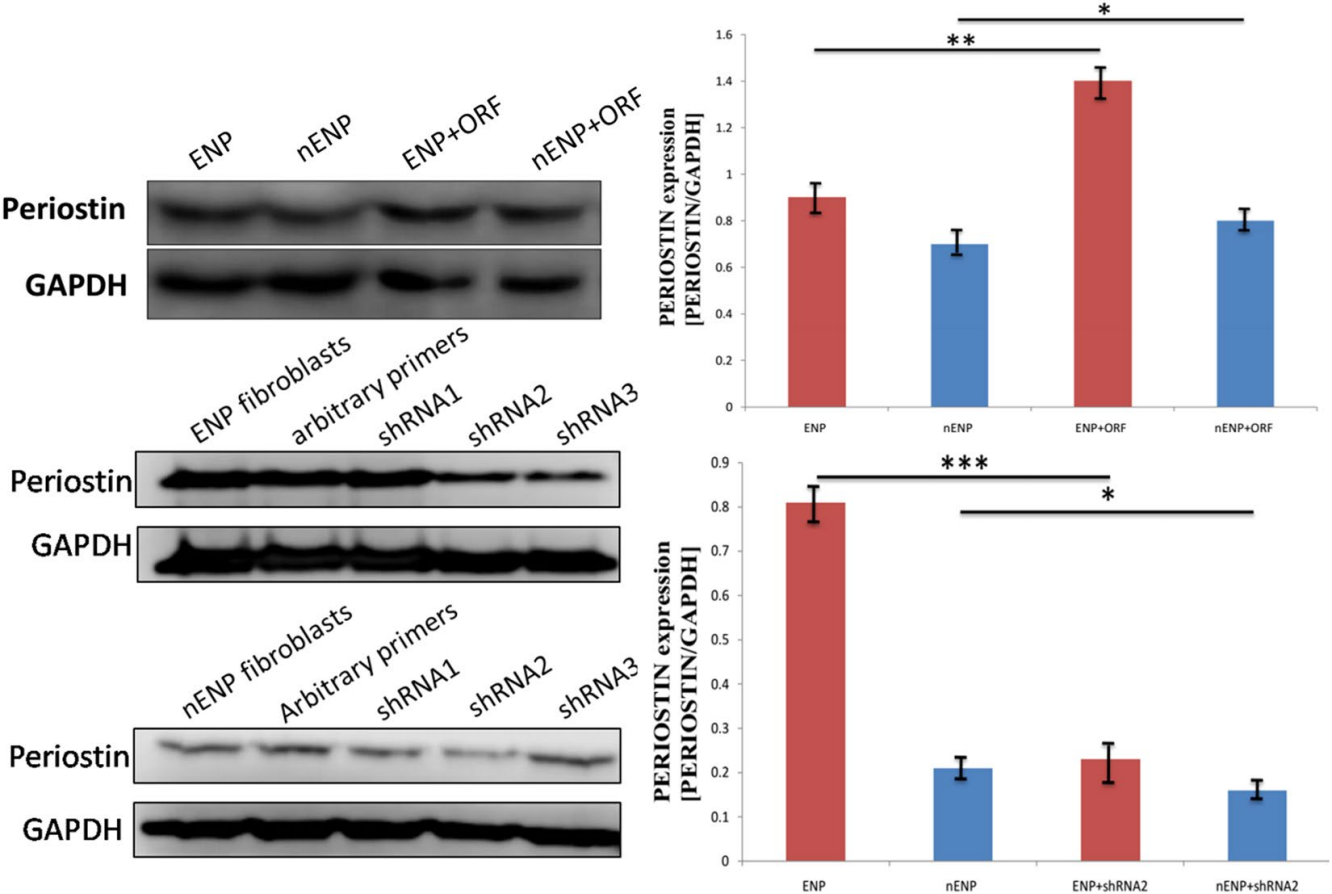
Western blot analysis of periostin protein in periostin overexpression and shRNA mediated knockdown NPDFs. *P < 0.05; n.s., not significant. shRNA, short hairpin RNA; ORF, open reading fragment.

PLKO-U6-periostin-shRNA-GFP and plko-u6-scramble were transfected into 293 T cells with the Lipofectamine 2000 Reagent (Invitrogen) according to the transfection protocol provided by the manufacturer. The short hairpin RNA (shRNA) and open reading fragment (ORF) sequence is provided in Table 2. The virus supernatant (2 ml) was collected at 48 h and 72 h and centrifuged, and the multiplicity of infection (MOI) of 10^−5^ was used for initial infection to infect the target cells for 72 h. The expression level of periostin was detected using western blotting (Fig. 1). The knock down of periostin (periostin^KD^) involved the periostin shRNA2, and the overexpression of periostin (periostin^OE^) was the periostin ORF.

**Table 2:** shRNA and ORF sequence.

Primer-template	Sequence
Periostin shRNA1	F: ccggacttgggaacaggactttatactcgagtataaagtcctgttcccaagttttttg
	R: aattcaaaaaacttgggaacaggactttatactcgagtataaagtcctgttcccaagt
Periostin shRNA2	F: ccggcgagccttgtatgtatgttatctcgagataacatacatacaaggctcgtttttg
	R: aattcaaaaacgagccttgtatgtatgttatctcgagataacatacatacaaggctcg
Periostin shRNA3	F: ccggggcatactttggaatccattactcgagtaatggattccaaagttagctttttg
	R: aattcaaaaaggctaactttggaatccattactcgagtaatggattccaaagttagcc
Periostin ORF sequence	F: ccgctcgaggccaccatgattccctttttacccatg
	R: cggaattctcactgagaacgacctccct
Scramble sequence	F: ccggcaacaagatgaagagcaccaactcgagttggtgctcttcatcttgttgtttttg
	R: aattcaaaaacaacaagatgaagagcaccaactcgagttggtgctcttcatcttgttg

### Statistical analysis

The statistical analysis was performed using SPSS 19.0 (SPSS Inc, Chicago, IL, USA). All of the values were presented as the mean ± standard deviation (SD). The data were analyzed by Chi-squared test and Student t test where appropriate. *P* < 0.05 was considered statistically significant.

## Results

### Periostin and VEGF are differentially expressed between the ENP and nENP subtypes

**Figure 2:**
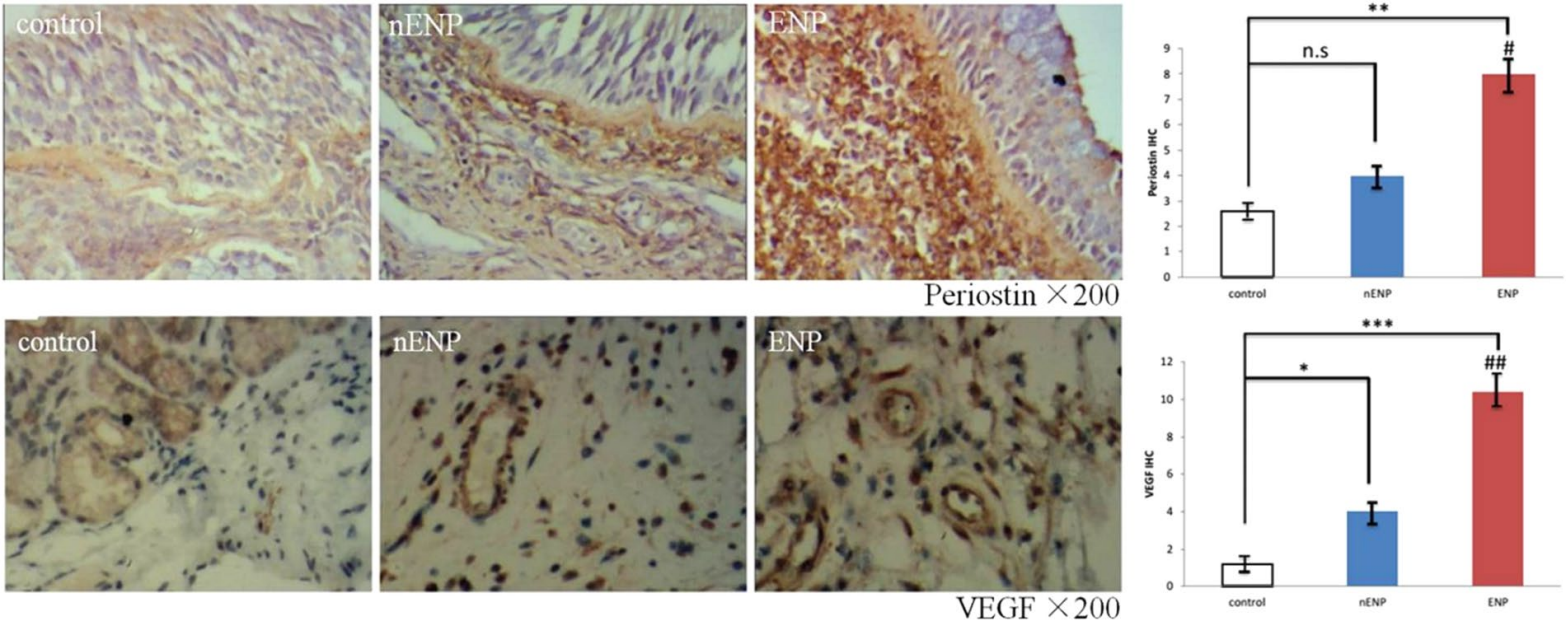
Immunohistochemistry of periostin and VEGF in NP and controls. Periostin (upper panel) is expressed in the extracellular matrix, and the thickened basement membrane in the ENP. The positive expression of VEGF (lower panel) is mainly distributed in vascular endothelial cells, glandular cells, and fibroblast cells. Magnification: ×200. Students t test was used to calculate statistical significance. *P < 0.05, **P < 0.01, ***P < 0.001 compared with control. ^#^ *P* < 0.05, ^##^ *P* < 0.01, nENP vs. ENP.

**Figure 3:**
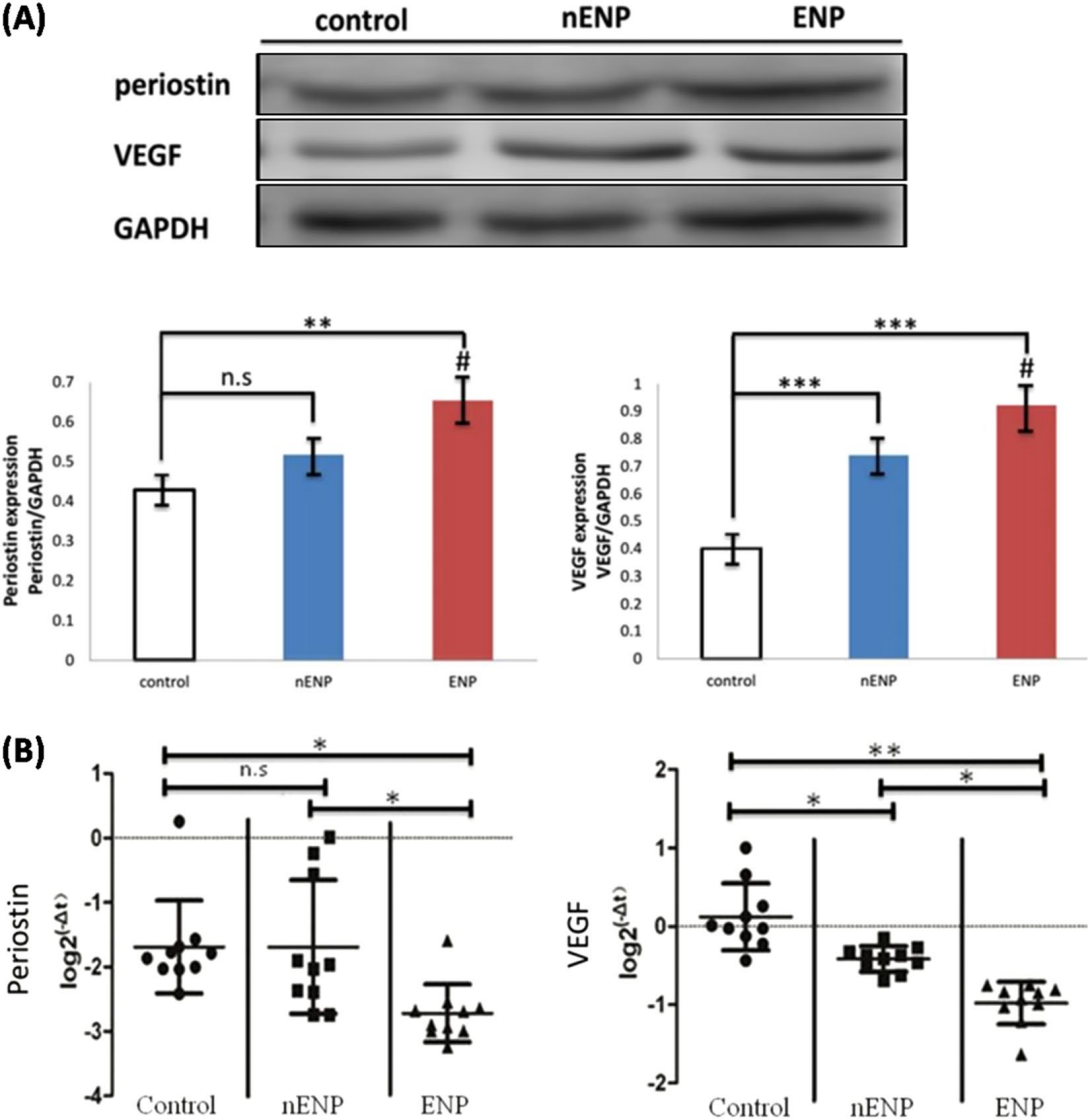
Expressions of periostin and VEGF in nasal polyps and controls. (**A**) Western blot analysis of periostin and VEGF in NPs and controls. Student t test was used to calculate statistical significance. **P < 0.01, ***P < 0.001 compared with control; ^#^ *P* < 0.05, nENP vs. ENP. (**B**) qPCR analysis of periostin and VEGF mRNA levels in nasal polyps and controls. *P < 0.05; **P < 0.01. Student t test was used to calculate statistical significance. *P < 0.05; **P < 0.01; n.s., not significant.

The histologic profile of polyp tissues in patients with ENP and nENP and controls was characterized by evaluating submucosal capillary vessels. The mean number of submucosal capillary vessels was 10.1 ± 2.77 in the ENP group compared with 5.8 ± 1.91 (*P* < 0.01) in the nENP group and 3.7 ± 1.61 (*P* < 0.01) in the control subjects. Accordingly, the staining intensity of periostin and VEGF was significantly higher in ENP tissues than in nENP tissues and controls (Fig. 2). Further, we found that the polyp tissues from the ENP group exhibited significantly higher periostin and VEGF mRNA and protein levels than those from the nENP group and control subjects (Fig. 3). Collectively, these findings demonstrate that the inflammatory responses differ between ENP and nENP.

### Periostin regulates VEGF, RANTES and eotaxin-2 expression in NPDFs *in vitro*

**Figure 4:**
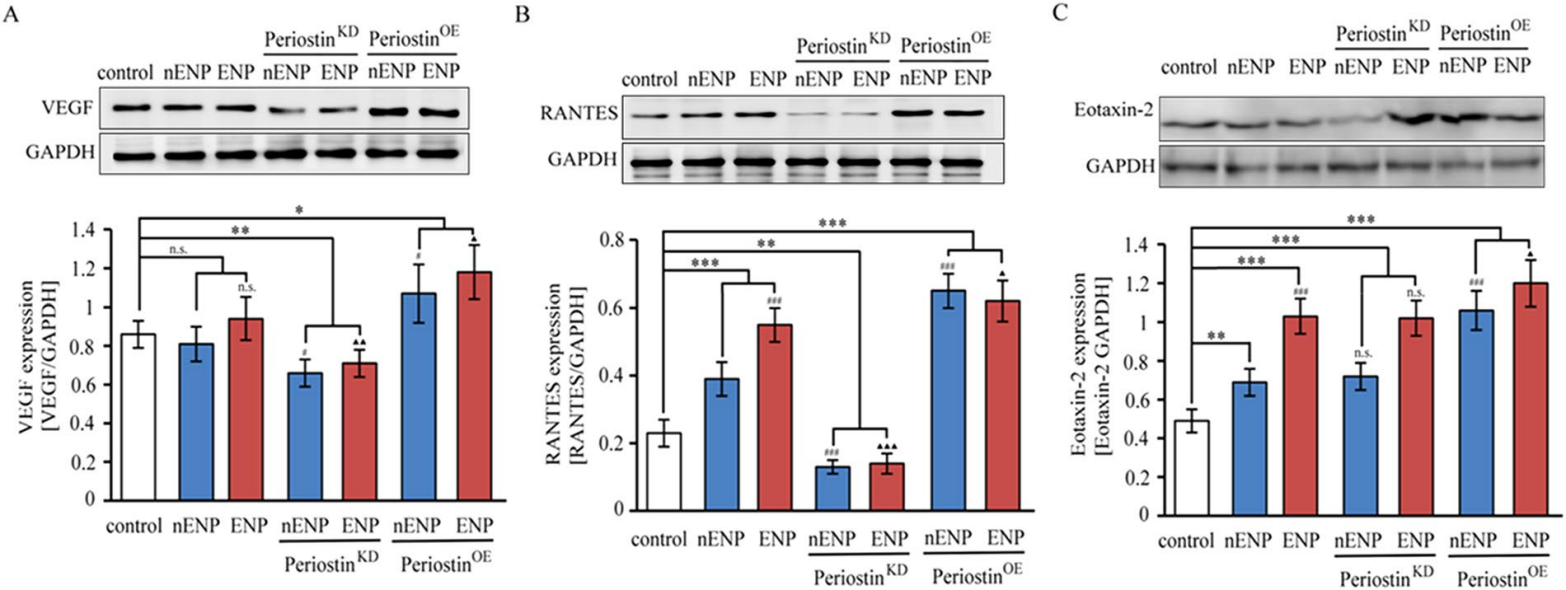
Periostin is important for the induction of VEGF, RANTES and eotaxin-2 in NPDFs. Western blot analysis of (**A**) VEGF, (**B**) RANTES and (**C**) eotaxin-2 expression in NPDFs. Students t test was used to calculate statistical significance. *P < 0.05; **P < 0.01; ***P < 0.001 compared with control. ^▲^ *P* < 0.05; ^▲▲^ *P* < 0.01; ^▲▲▲^ *P* < 0.001 compared with ENP. ^#^ *P* < 0.05, ^###^ *P* < 0.001 compared with nENP. n.s., not significant. periostin^OE^, periostin overexpression; periostin^KD^, periostin knock down.

To gain insights into the role of periostin in different subtypes of NP, we expressed and repressed periostin in NPDFs derived from NP. Periostin protein levels were detected by Western blot. Increases of 1.59-fold and 1.35-fold were observed in the periostin^OE^ eosinophilic NPDFs and periostin^OE^ non-eosinophilic NPDFs, respectively, and decreases of 3.72-fold and 1.84-fold were observed in the periostin^KD^ eosinophilic NPDFs and periostin^KD^ non-eosinophilic NPDFs, respectively (Fig. 1). We continued examining the effects of periostin expression on VEGF, RANTES and eotaxin-2 expressions in NPDFs *in vitro*. We observed that fostered periostin expression significantly up-regulated the expression of VEGF, RANTES and eotaxin-2 protein in the NPDFs (Fig. 4). In contrast, periostin knockdown in NPDFs significantly reduced the expression of VEGF and RANTES protein, but not eotaxin-2 in the NPDFs (Fig. 4). However, no significant differences in the protein expressions of VEGF, RANTES were observed between the NP subtype groups (Fig. 4A and B). Additionally, the protein expression of eotaxin-2 was higher in the eosinophilic NPDFs and periostin^KD^ eosinophilic-NPDFs compared with non-eosinophilic NPDFs and periostin^KD^ non-eosinophilic NPDFs respectively. But there was no significant difference between the periostin^OE^ NPDFs groups (Fig. 4C). These findings suggest that periostin might exert regulatory mechanisms on eosinophil recruitment and microvascular remodelling by inducing VEGF, RANTES and eotaxin-2 expression.

### Periostin knockdown inhibits the production of RANTES by TNF-α stimulated eosinophilic-NPDFs

**Figure 5:**
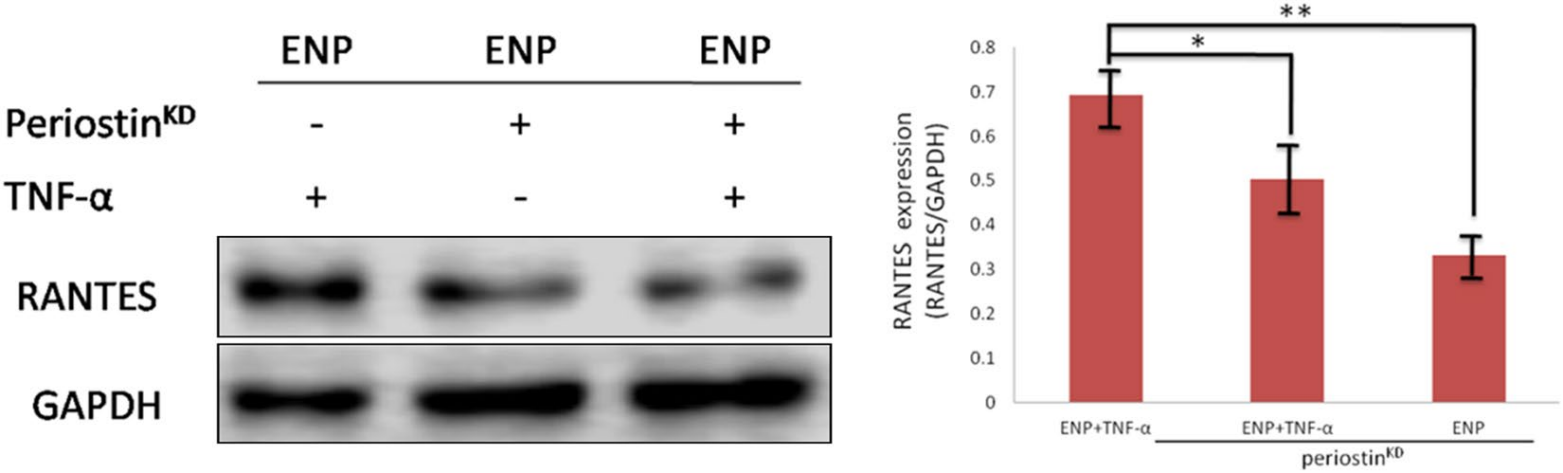
Periostin knockdown inhibits the production of RANTES by TNF-α stimulated eosinophilic-NPDFs. Eosinophilic NPDFs were subjected to TNF-α as indicated for 72 hours, then RANTES protein levels were evaluated by Western blot. Student t test was used to calculate statistical significance. *P < 0.05; **P < 0.01. periostin^KD^, periostin knock down.

To elucidate the underlying mechanism by which the induction of RANTES, which recruit eosinophils, is down-regulated in periostin^KD^ eosinophilic NPDFs, we investigated the expression of RANTES by PN-deficient fibroblasts in response to TNF-α. Upon stimulation with TNF-α (20 ng/ml for 72 hours), the expression of RANTES was markedly down-regulated in periostin^KD^ eosinophilic NPDFs with TNF-α stimulation compared with periostin-independent eosinophilic NPDFs without TNF-α stimulation (Fig. 5). These results suggest that the impaired production of RANTES by fibroblasts in response to TNF-α could at least partly explain the underlying mechanism of reduced eosinophil recruitment in PN-deficient ENP.

## Discussion

The present study demonstrated that the protein and mRNA levels of periostin and VEGF were higher in human ENP tissue than in nENP and control tissues. In addition, the periostin protein level was higher in primary eosinophilic-NPDFs than in non-eosinophilic-NPDFs or control subject fibroblasts. An up- or down-regulated periostin level could promote or inhibit the expression of VEGF and RANTES in NPDFs. However, we found that periostin regulated the protein expression of VEGF and eotaxin-2, although the differences were weak. These findings broaden our understanding of the pathophysiology underlying ENP.

ENP is characterized by the eosinophil accumulation and tissue oedema in the sinus mucosa and nasal polyps^19^. It is this distinct histopathology of ENP that has resulted in an increased interest in using individual therapeutic approaches to the disease^21^. However, little is known regarding the development of these diseases. To clarify the mechanisms underlying ENP, many cytokines in ENP tissues have been investigated, and higher concentrations of eotaxin-2^22^, RANTES^23^, serum IgE^24^, and p63/p73^25^ have been detected in patients with ENP than in patients with nENP or in controls. However, the role of these cytokines in the inflammatory process underlying ENP requires further exploration. Recently, several studies have determined that the expression of VEGF was significantly higher in nasal polyps than in the middle turbinate from controls^13, 26, 27^. In this study, we further observed periostin and VEGF immunoreactivity, and the mRNA and protein expression levels were markedly increased in ENP. This differential expression of periostin and VEGF might be associated with distinct features in the tissue remodelling of ENP.

Periostin is a 90-kDa secreted protein that has been recognized as a matricellular protein, and the number of studies involving periostin has grown rapidly in recent decades. Periostin was reported to have an important role in wound repair and the epithelial-mesenchymal transition of cancer cells^28–30^. Additionally, an allergen challenge could increase periostin expression in the airway epithelium, subepithelium, smooth muscle, and inflammatory cells^31^, and periostin has been shown to act on eosinophils and enhance their recruitment to lesions^32^. In our recent study, we found that ENPs were associated with Th2-mediated allergic disease and allergen sensitization^18^. The periostin-rich microenvironment develops in areas that are associated with insult, and exposure to allergens in atopic diseases can be considered an insult^33^. The overexpression of periostin in ENP, which might be induced by an allergen challenge, has been thought to take part in the severe epithelial damage in the nasal mucous membranes, and the continuous and longstanding overexpression of periostin contributed to persistent and progressive inflammation locally accompanied by extracellular matrix accumulation and inflammatory cell infiltration^33^. These findings strongly support the suggestion that periostin is produced locally in the sinonasal tissues and contributes to polyp formation.

Angiogenesis plays a vital role in polyp initiation and progression, and angiogenesis and microvascular remodelling are elements of tissue remodelling in nasal polyps that depend on a coordinated balance of the levels of VEGF^34^. In the histopathological study of ENP tissues, we found that the mean number of submucosal capillary vessels was increased. However, the association between periostin and VEGF in NP tissue remains unclear. To address this issue, the present study examined the possible mechanisms underlying the action of periostin in NPDFs, and we found that the expression of VEGF protein was increased significantly in the periostin^OE^ NPDFs compared with control subjects. These results suggested that periostin could act on NPDFs to produce VEGF expression and promote angiogenesis indirectly. Moreover, VEGF, by increasing microvascular permeability, allows the extravasation of plasma proteins and inflammatory cells that participate in the accumulation of the extracellular matrix, thereby accelerating NP growth^35^. One characteristic of NP is substantial tissue oedema. This oedema has been attributed to local VEGF production, which plays an important role in angiogenesis and modulating capillary permeability^36, 37^, suggesting that higher concentrations of VEGF might lead to distinct histological features in ENP compared with nENP and control subjects.

Tissue eosinophilia is considered a hallmark of NP and a risk factor for disease recurrence^38, 39^. However, the question remains why eosinophils accumulate in ENP tissue. Further investigation revealed that the expression of RANTES and eotaxin-2 protein in the ENP and nENP could be mediated by periostin, with no significant difference in the 2 NP subtypes. These findings suggest that RANTES and eotaxin-2, which are released locally in relation to periostin concentration, may induce eosinophils to migrate out from the vasculature into sites of inflammation. Consequently, increased expression of periostin in tissues is associated with eosinophilic inflammation and tissue oedema.

To determine the potential therapeutic effect of periostin^KD^ in inhibiting angiogenesis and eosinophil recruitment in ENP, we found that periostin^KD^ could inhibit VEGF and RANTES expression in NPDFs. Thus, we investigated RANTES protein expression by periostin^KD^ fibroblasts in response to TNF-α, a potent inducer of chemokine production^40^ and TNF-α might induce RANTES production through mediating expression of periostin^41, 42^. Furthermore, TNF-α failed to effectively stimulate the production of RANTES protein in the periostin^KD^ eosinophilic-NPDFs compared with periostin-independent eosinophilic-NPDFs. Given that RANTES and VEGF promote eosinophil recruitment and microvascular remodelling into the polyp microenvironment, the effect of silencing periostin expression against ENP occurrence and proliferation might be mediated by the inhibition of RANTES and VEGF expression. Accordingly, our findings indicate that periostin is a potential therapeutic agent specifically for ENP. Further studies with *in vivo* experiments will be summarized in our next study.

In addition, the smoking rate of patients in the ENP group was significantly higher than in the nENP group in our study cohort. Thus, whether the expression of periostin is associated with cigarette smoke exposure requires further exploration.

In conclusion, the present study revealed that periostin might promote angiogenesis and eosinophil recruitment, indicating that periostin is a critical matricellular component in remodelling the tissue microenvironment in ENP. These findings show that periostin might enhance the occurrence and progression of ENP and be a potential target in the therapeutic intervention for ENP.

## Electronic supplementary material

supplementary figures
