# Supplementary material for: The Role of Periostin in the Occurrence and Progression of Eosinophilic Chronic Sinusitis with Nasal Polyps
Source: Sci Rep. 2017 Aug 25;7:9479. doi: 10.1038/s41598-017-08375-2 (PMC5572682; doi:10.1038/s41598-017-08375-2)

The Role of Periostin in the Occurrence and Progression of Eosinophilic Chronic Sinusitis with Nasal Polyps

Ming Xu[1,3,*], Daishi Chen[1,*], Haojie Zhou[2,*], Weiwei Zhang[3,*], Jun Xu[3] **&** Lei Chen[1]

1.Department of Otorhinolaryngology head and neck surgery, Chinese PLA General Hospital, Beijing 100083, China
2. Ningbo Diagnostic Pathology Center, Ningbo 315021, China
3. Department of Otorhinolaryngology, the Affiliated Hospital of The Medical School of Ningbo University, Ningbo 315020, China

Suppl. Fig. 1

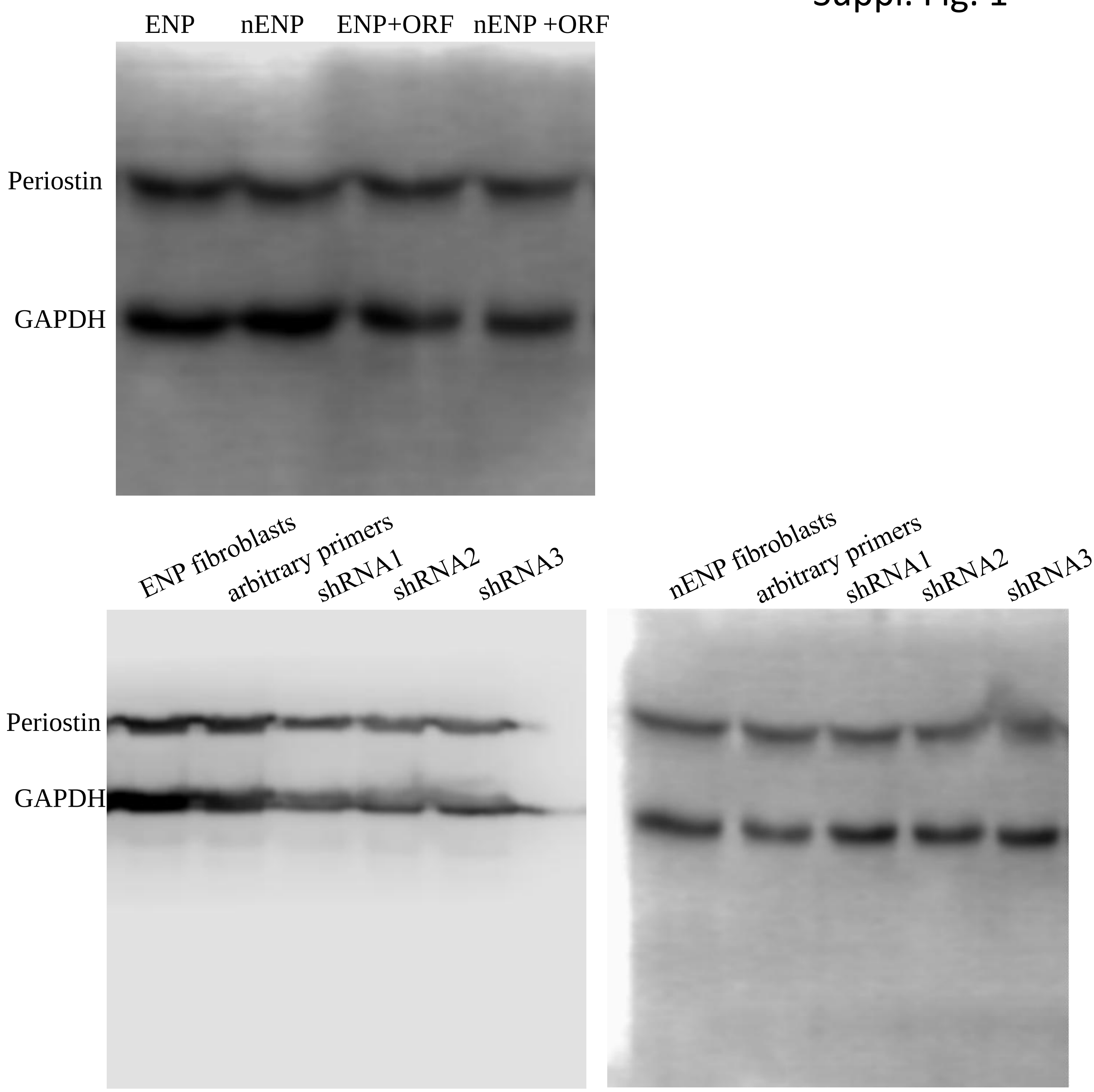

After fibroblasts were transfected with lentivirus harbouring periostin shRNA2 and periostin ORF periostin protein levels were determined by using Western blot analysis.

Suppl. Fig. 3

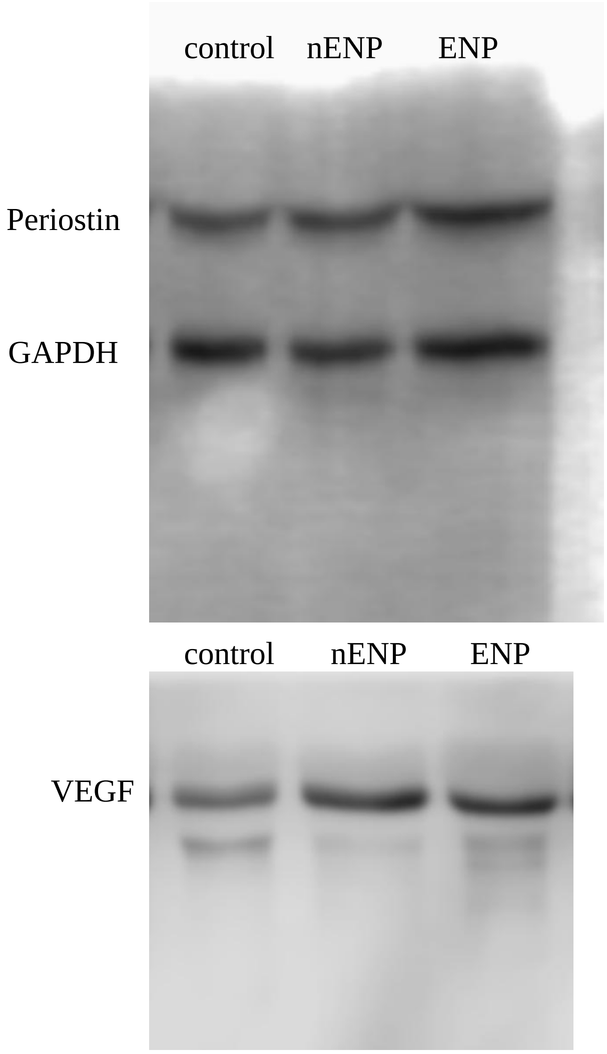

Western blot analysis of periostin protein in periostin overexpression and shRNA mediated knockdown NPDFs. *P< 0.05; n.s, not significant. shRNA, short hairpin RNA; ORF, open reading fragment.

Suppl. Fig. 4

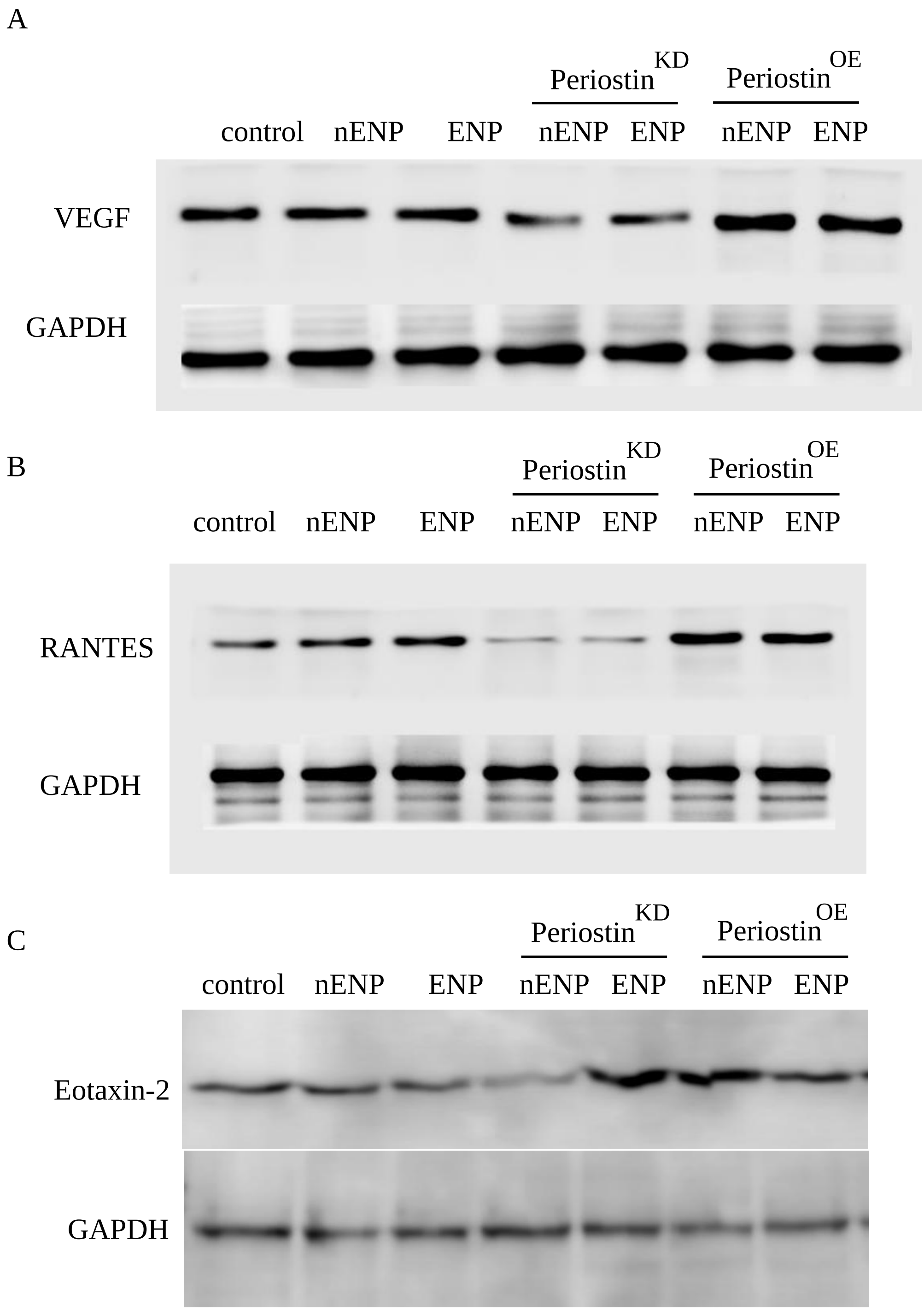

Periostin is important for the induction of VEGF, RANTES and eotaxin-2 in NPDFs. Western blot analysis of (A) VEGF, (B) RANTES and (C) eotaxin-2 expression in NPDFs. Students t test was used to calculate statistical significance. * $P < 0.05$; ** $P < 0.01$; *** $P < 0.001$ compared with control. ▲$P< 0.05$; ▲▲$P< 0.01$; ▲▲▲$P< 0.001$ compared with ENP. # $P < 0.05$, ### $P < 0.001$ compared with nENP. n.s, not significant. periostin^{OE}, periostin overexpression; periostin^{KD}, periostin knock down.

Suppl. Fig. 5

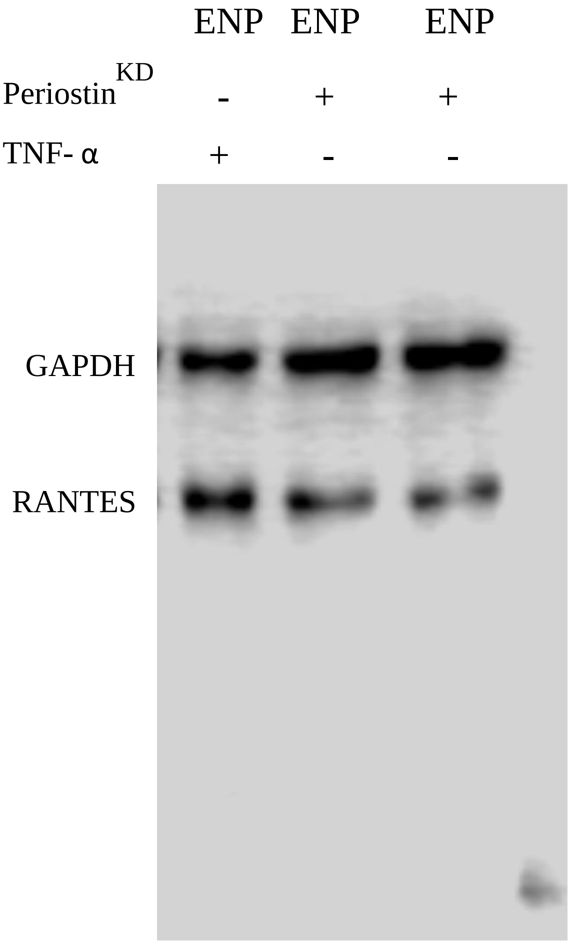

Periostin knockdown inhibits the production of RANTES by TNF-α stimulated eosinophilic-NPDFs. Eosinophilic NPDFs were subjected to TNF-α as indicated for 72 hours, then RANTES protein levels were evaluated by Western blot. Student t test was used to calculate statistical significance. * $P < 0.05$; ** $P < 0.01$. periostin^{KD}, periostin knock down.

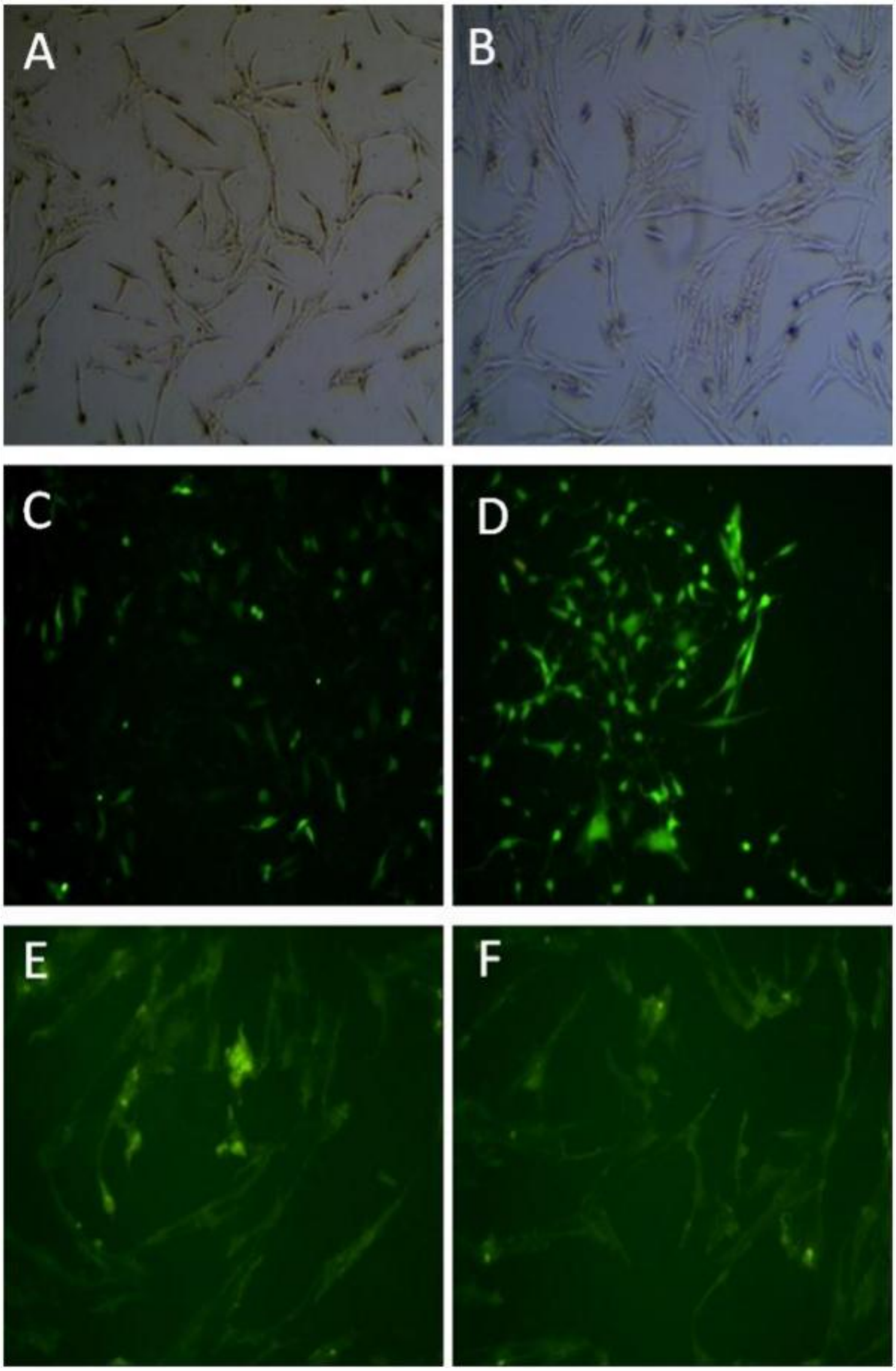

Fluorescence microscopy of periostin shRNA and periostin ORF under microscopy and fluorescence microscopy. The effective rate of transduction was more than 90% transfection prior to each experiment (n=5)(Fig. 2.B, P<0.05). A. nENP fibroblasts; B. ENP fibroblasts; C. nENP fibroblasts after transfection with PN shRNA2 under fluorescence microscopy; D. ENP fibroblasts after transfection with PN shRNA2 under fluorescence microscopy; E. nENP fibroblasts after transfection with PN ORF under fluorescence microscopy; F. ENP fibroblasts after transfection with PN ORF under fluorescence microscopy.

Privious Fig.3.B

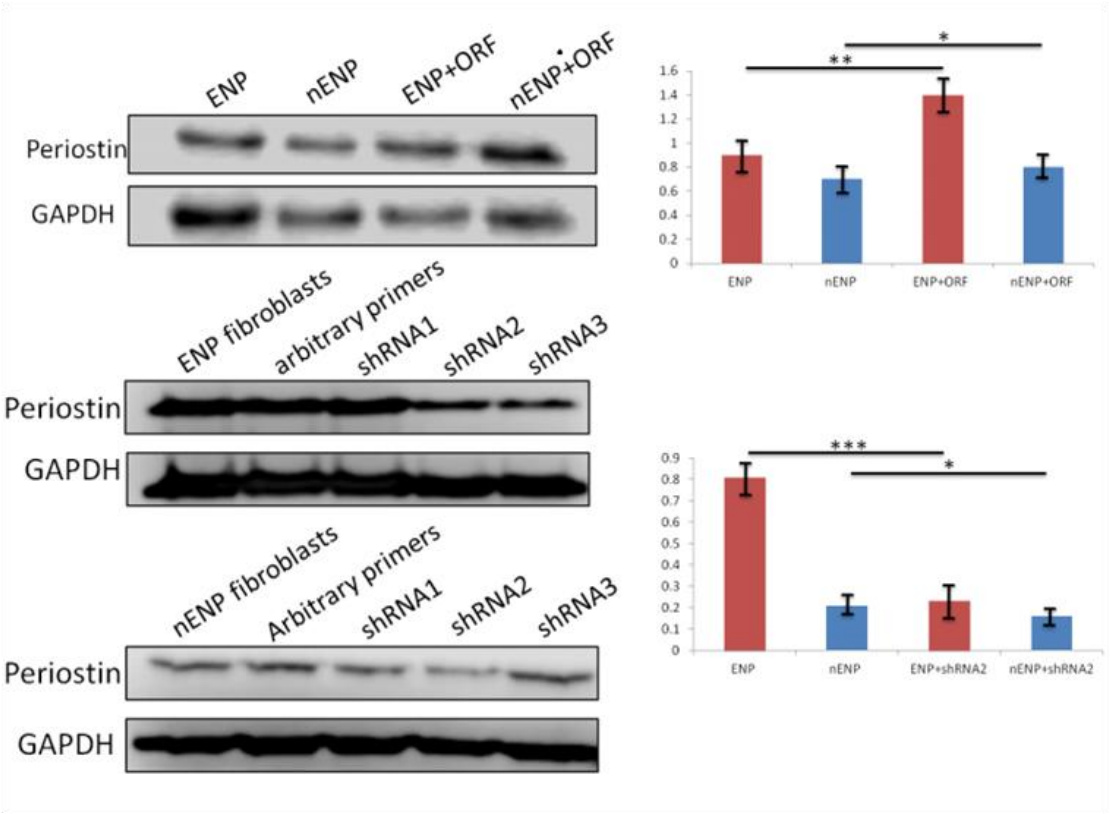

Privious Fig.4

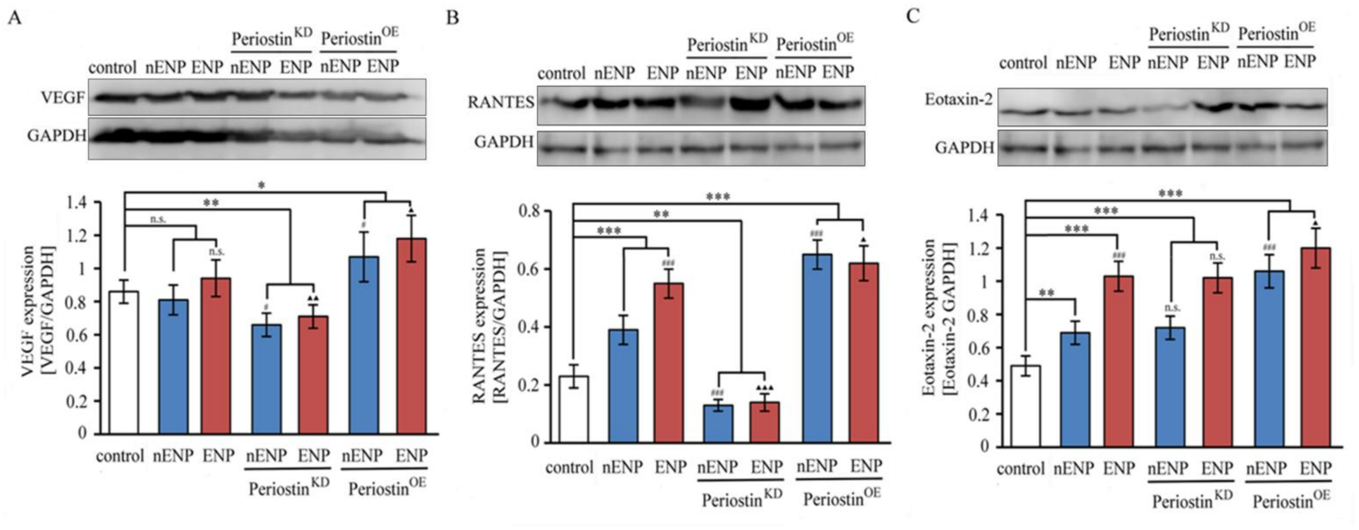

Supplement: Supplementary file 1 — supplementary figures [file 41598_2017_8375_MOESM1_ESM.pdf]
